# Extended trochanteric osteotomy is a safe procedure in two-stage hip revision: a systematic review of the literature

**DOI:** 10.1007/s00590-023-03497-y

**Published:** 2023-02-27

**Authors:** Giorgio Cacciola, Fortunato Giustra, Francesco Bosco, Alessandro Aprato, Federico De Meo, Pietro Cavaliere, Daniele Vezza, Matteo Giachino, Luigi Sabatini, Alessandro Massè

**Affiliations:** 1 Department of Orthopaedics and Traumatology, University of Turin, CTO, Via Zuretti 29, 10126 Turin, Italy; 2 Istituto Ortopedico del Mezzogiorno d’Italia “Franco Scalabrino”, Via Consolare Pompea, 98100 Messina, Italy; 3 Department of Orthopaedics and Traumatology, Ospedale San Giovanni Bosco - ASL Città di Torino, Turin, Italy

**Keywords:** Extended trochanteric osteotomy, ETO, Periprosthetic joint infection, PJI, Two-stage revision

## Abstract

**Background:**

Extended trochanteric osteotomy (ETO) has proved to be an effective technique in complicated stem removal in femoral aseptic loosening or periprosthetic fracture. Debate remains about its safety in periprosthetic joint infection (PJI). The primary aim of this study is to analyze the ETO reinfection and union rate in two-stage hip revision.

**Material and methods:**

A systematic literature review was performed regarding all studies reporting ETO outcomes in the two-stage revision for hip PJI up to October 2022, according to the Preferred Reporting Items for Systematic Reviews and Meta-Analyses criteria. A literature search was conducted in the following databases: MEDLINE/EMBASE, Scopus, Web of Science, and Cochrane. Quality assessment of the articles was performed using the Methodological Index for Non-Randomized Studies. This systematic review was registered in the International Prospective Registry of Systematic Reviews. Patient demographic, clinical, and surgical data were collected.

**Results:**

This systematic review included and analyzed nine clinical studies with a total of 382 ETO PJI hips in two-stage revision. The overall ETO reinfection rate was 8.9% (34 hips), consistent with the reinfection rate after two-stage revision in patients without ETO. The overall ETO union rate was 94.8% (347 hips), comparable to the ETO union rate in non-septic patients. Compared between a group of patients with ETO PJI and a group of patients with non-PJI ETO, there were no significant differences in postoperative complications, both septic and aseptic, and for postoperative HHS.

**Conclusion:**

ETO proved to be a safe and effective procedure in PJI revisions. It may be a viable option in challenging femoral stem removal during the two-stage hip revision in PJI.

**Level of evidence:**

IV.

## Introduction

Extended trochanteric osteotomy (ETO) is a well-validated surgical procedure characterized by a proximal femur osteotomy to facilitate a stable stem extraction while reducing periprosthetic risk fracture [1–3]. ETO allows cement or metal hardware removal through femoral canal access and correcting lower limb length discrepancies or femoral rotation defects [4].

Several publications demonstrated that ETO is a safe and effective procedure in aseptic revision [3–5]. Malahias et al. [6], in their systematic review, reported an osteotomy flap healing rate of 93.1 percentage (%), significant stem subsidence greater than 5 mm in 7.1% of cases, and a postoperative complication rate of 8.1% from a total of 1378 ETOs executed for aseptic revision total hip arthroplasty (THA).

ETO may also be performed in the first stage of a two-stage hip revision for periprosthetic joint infection (PJI) treatment; however, its application is controversial [7–9]. Despite numerous advantages, such as easier femoral stem extraction, better medullary canal exposure to facilitate debridement, and eventual cement or other metal hardware removal [8], important concerns persist. First, cables or cerclages used to fix the osteotomy flap could increase the reinfection rate; in addition, the presence of infection and eventual flap reopening during the reimplantation could raise flap nonunion risk [9].

The primary purposes of this systematic review are to evaluate safety in terms of recurrent infection, intraoperative fractures incidence, osteotomy flap healing rate and time in patients with PJI in whom ETO was performed during resection arthroplasty. Secondary aims include postoperative complications such as dislocation, fracture or loosening and patient-reported outcome scores (PROMs).

## Materials and methods

### Study design and methodology

A systematic review of the current literature up to October 1, 2022, according to the Preferred Reporting Items for Systematic Reviews and Meta-Analyses (PRISMA) criteria [10, 11], was conducted for studies in which ETO was performed as part of a two-stage revision for hip PJI [12]. A search of the US national library of Medicine (MEDLINE/EMBASE), Scopus, Web of Science, and Cochrane Database of systematic reviews databases with the following key terms in association with the Boolean operators AND, OR: “extended trochanteric osteotomy,” “ETO,” “transfemoral approach,” “periprosthetic joint infection,” and “PJI.”

### Inclusion and exclusion criteria

Two independent authors (GC and FB) reviewed the titles and abstracts of the literature, searching for relevant articles on ETO in patients with hip PJI. A third author (FG) was consulted to resolve any doubts. Titles and abstracts of full-text articles were reviewed for inclusion and exclusion criteria. Studies that met the following criteria were included: studies written in English, original clinical studies investigating the outcomes of ETO in hip PJI, studies with at least ten patients, and studies that clearly provide the rate of recurrence of infection and the rate of recovery of ETO. Studies written in non-English, case reports, preclinical studies, book chapters, technical reports, editorials, biomechanical reports or literature reviews, studies using ETO in non-PJI patients, studies that do not clearly report the reinfection rate or union rate of ETO were excluded from the analysis. Studies published after October 2022 were also excluded from the analysis.

### Search strategy and study screening

**Fig. 1:**
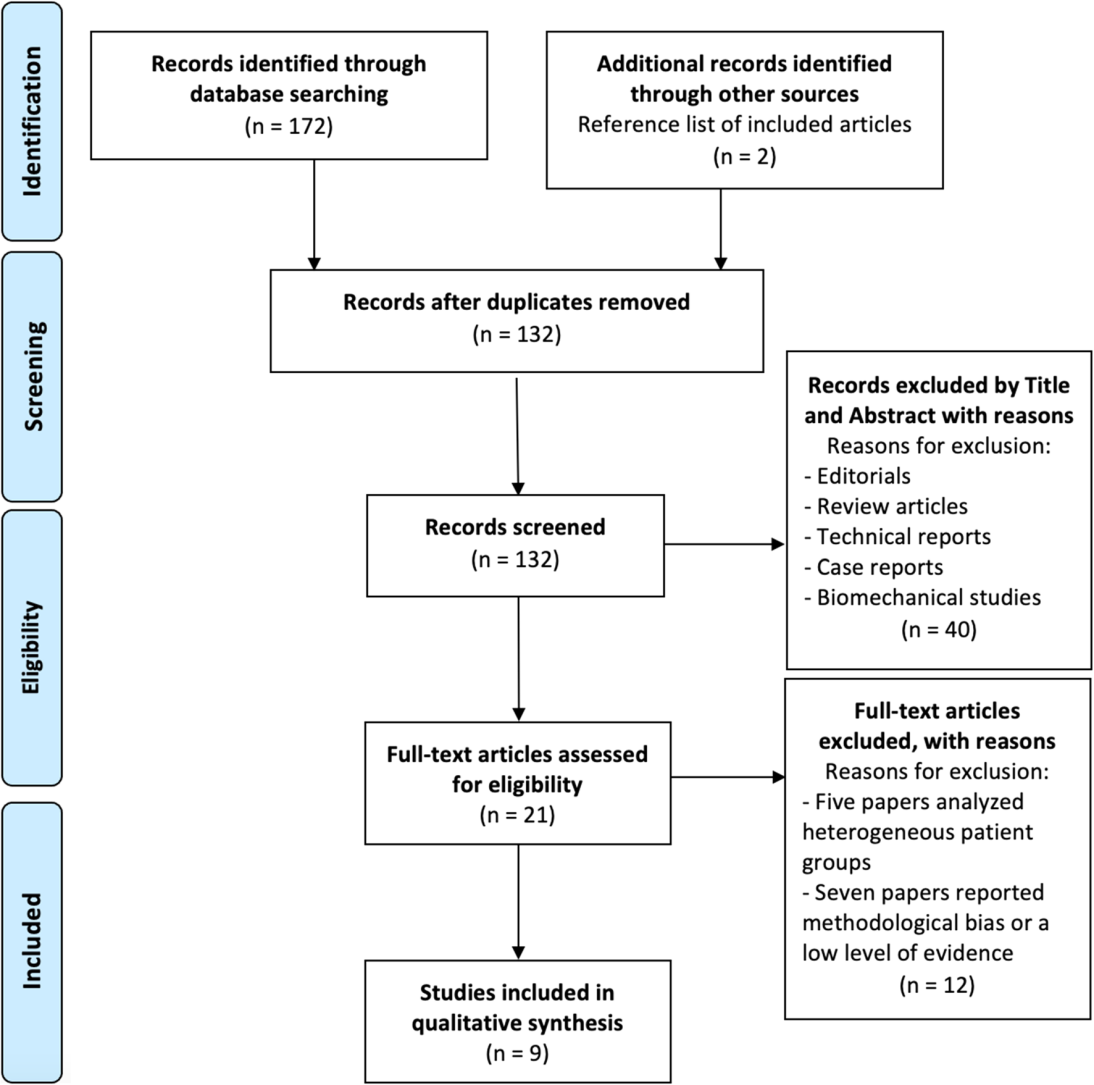
Preferred reporting items for systematic review and meta-analysis (PRISMA) flow diagram

A total of 172 studies were identified. After the exclusion of duplicates, 132 studies were examined. After title and abstract screening, 21 clinical studies were assessed for full-text evaluation, and nine clinical studies [7–9, 13–18] that met the inclusion criteria were finally included in this systematic review. The bibliography of each article was cross-checked to find other relevant publications for inclusion in the current study. The PRISMA flowchart for study selection and search strategy is shown in Fig. 1 [10, 11].

### Methodological quality assessment

Each article included in this systematic review was evaluated following the Oxford Centre for Evidence-Based Medicine criteria [19]. Methodological Index for Non-Randomized Studies (MINORS) score [20] was used by two authors (GC and FB) to analyze the included studies' quality. A third author (FG) was consulted to resolve any additional uncertainties. This systematic review was registered in the International Prospective Register of Systematic Reviews (PROSPERO), CDR: CRD42022364300 [21, 22].

### Data collection

Two independent authors (GC and FG) performed data extraction of relevant studies that met the inclusion criteria. In addition, study data (first author, year of publication, study design, and quality), demographic data (number of hips, patients lost to follow-up or deceased, mean age at surgery, gender), and clinical data (reinfection rate, osteotomy flap healing rate and healing time, postoperative non-septic complications, and PROMs) were collected.

### Statistical analysis

Statistical analysis was performed with R software, version 4.0.5 (2020; R Core Team, Vienna, Austria). Descriptive statistical analysis was performed for all data obtained from the included studies. Continuous variables were calculated using mean values with a measure of variability as a range (minimum–maximum) or standard deviation (SD). Categorical variables were evaluated using absolute number and frequency distribution. A P value less than or equal to 0.05 was considered statistically significant.

## Results

### Included studies

A total of 387 ETO PJI hips were initially included in the analysis. After excluding patients who died from causes unrelated to surgical treatment or patients with data missing or lost to follow-up, 382 ETO PJI hips were included in the final analysis. There were 193 (50.5%) women, with a mean age of 64.9 (52.6–71.3) years. The mean time of follow-up was 4.7 (2–5.7) years. The results of methodological quality were performed with the MINORS score, and the results are reported in Table 1.

**Table 1:** Study characteristics and patient demographics

Author and publication year	LoE	MINORS score	N° of hips, initial cohort/final cohort	N° of hips lost to follow-up and/or died	Age	Gender female	Follow-up
			N°/N°	N° (%)	y.o., Mean ± SD/(range)*	N° (%)	Years, mean ± SD/(range)*
Morshed et al. 2005 [7]	IV	6	13/13	0 (0%)	52.6 (40–82.2)	5 (38.5%)	2 ± 1.1
Levine et al. 2009 [8]	IV	8	23/23	0 (0%)	61.7 (30–85)	15 (60%)	4.1 (2–7)
Lim et al. 2011 [9]	III	16	23/23	0 (0%)	58.5 (33–79)	12 (52.2%)	5.3 (2–10.3)
Fink et al. 2016 [13]	IV	9	81/76	5 (6.2%)	70.7 ± 9.8	37 (48.7%)	4.3 (2–9.8)
Petrie et al. 2017 [14]	IV	8	102/102	0 (0%)	67 (33–83)	56 (54.9%)	5.7 (0.1–14.4)
Shi et al. 2019 [15]	III	12	48/48	0 (0%)	59.3 (30–78)	19 (39.6%)	6 (2.1–11.8)
Hardt et al. 2021 [16]	III	16	32/32	0 (0%)	71.3 ± 10.5	21 (65.6%)	5.5 (3.2–7.5)
Lancaster et al. 2021 [17]	III	12	16/16	0 (0%)	63 ± 9	11 (68.8%)	2
Whittaker et al. 2022 [18]	III	16	49/49	0 (0%)	62 (32–86)	19 (38.8%)	2.9 (0.1–12.1)

N°: number of evaluation cases; LoE: levels of evidence; MINORS score: Methodological index for non-randomized studies score; %: percentage; SD: standard deviation; y.o.: years old; *: if SD was not reported, values range were recorded

### Rate and time of union and reinfection rate of ETO

Eight studies reported the ETO union rate [7–9, 13–16, 18]. An average ETO union rate after two-stage revision for hip PJI of 94.8% was reported. The union rate ranges from a lower mean of 87.3% [14] to a mean of 100% reported in three studies [7, 9, 18]. Five studies reported the ETO union time [7–9, 13, 15]. A mean ETO union time after a two-stage revision for PJI of the hip of 12 (10.6–15.6) weeks was reported. All nine studies [7–9, 13–18] reported the postoperative ETO reinfection rate in the setting of a two-stage revision for hip PJI. The overall reinfection rate was 8.9%. The rate ranged from an average of 2.9% [14] to an average of 23.1% [7] (Table 2).

**Table 2:** Union rate and time and reinfection rate of ETO

Author and publication year	N° of Hips	ETO union rate	ETO union time	ETO reinfection rate
	N°	N° (%)	Weeks, N°	N° (%)
Morshed et al. 2005 [7]	13	13 (100%)	15.6	3 (32.1%)
Levine et al. 2009 [8]	23	22 (95.7%)	11.5	3 (13%)
Lim et al. 2011 [9]	23	23 (100%)	10.6	1 (4.3%)
Fink et al. 2016 [13]	76	75 (98.7%)	12	5 (6.6%)
Petrie et al. 2017 [14]	102	89 (87.3%)	N/A	3 (2.9%)
Shi et al. 2019 [15]	48	45 (93.8%)	12	2 (4.2%)
Hardt et al. 2021 [16]	32	31 (96.9%)	N/A	4 (12.5%)
Lancaster et al. 2021 [17]	16	N/A	N/A	3 (18.8%)
Whittaker et al. 2022 [18]	49	49 (100%)	N/A	10 (20.4%)
Overall	382	347 (94.8%)*	12**	34 (8.9%)

N°: number of evaluation cases; %: percentage; ETO: Extended Trochanteric Osteotomy; N/A: not available; *: Overall union rate ETO excluding cases from Lancaster et al. [17]. **: Overall union time ETO excluding cases from Petrie et al. Hardt et al. Lancaster et al. Whittaker et al. [14, 16–18]

### Postoperative complications

Eight studies reported postoperative complications [7–9, 13–16, 18]. The overall complication rate was 17.7%. The recurrent dislocation was the most frequent postoperative complication in 8.2% of cases (30 of 366 cases), followed by stem subsidence > 5 mm in 4.1%, periprosthetic fracture in 3% (11 of 366 cases), heterotopic ossification in 1.6%, aseptic loosening in 0.5%, and sciatic nerve palsy in 0.3% (Table 3).

**Table 3:** Non-septic complications and PROMs

Author and publication year	No° of hips	Dislocation	Periprosthetic fracture	Aseptic loosening	Sciatic Nerve palsy	Heterotopic ossification	Stem subsidence > 5 mm	HHS pre/post
	N°	N° (%)	N° (%)	N° (%)	N° (%)	N° (%)	N° (%)	N°/N°, mean
Morshed et al. 2005 [7]	13	4 (30.8%)	3 (6.1%)	1 (7.7%)	0 (0%)	6 (46.2%)	3 (23.1%)	25/68
Levine et al. 2009 [8]	23	2 (8.7%)	0 (0%)	0 (0%)	0 (0%)	0 (0%)	0 (0%)	N/A*
Lim et al. 2011 [9]	23	1 (4.3%)	2 (4.2%)	0 (0%)	1 (4.3%)	0 (0%)	1 (4.3%)	36.1/81.1
Fink et al. 2016 [13]	76	5 (6.6%)	4 (3.9%)	0 (0%)	0 (0%)	0 (0%)	5 (6.6%)	46.9/86.6
Petrie et al. 2017 [14]	102	4 (3.9%)	0 (0%)	0 (0%)	0 (0%)	0 (0%)	0 (0%)	n/a
Shi et al. 2019 [15]	48	4 (8.3%)	2 (8.7%)	0 (0%)	0 (0%)	0 (0%)	0 (0%)	30.2/85.7
Hardt et al. 2021 [16]	32	4 (12.5%)	0 (0%)	1 (3.1%)	0 (0%)	0 (0%)	4 (12.5%)	37.9/65.9
Whittaker et al. 2022 [18]	49	6 (12.2%)	0 (0%)	0 (0%)	0 (0%)	0 (0%)	2 (4.1%)	N/A
Overall	366	30 (8.2%)	11 (3%)	2 (0.5%)	1 (0.3%)	6 (1.6%)	15 (4.1%)	38.5/71.7

N°: number of evaluation cases; mm: millimeters; HHS: Harris Hip Score; N/A: not available; %: percentage; Pre: preoperative; post: postoperative; *Reported the mean preoperative and postoperative modified D'Aubigne and Postel scores from a mean preoperative of 2.4 for pain and 2.6 for ambulation to a mean postoperative of 5.3 for pain and 4.9 for walking

### PROMs

Five studies (192 cases) reported the mean preoperative and postoperative Harris Hip Score (HHS) [7, 9, 13, 15, 16]. The mean preoperative HHS was 38.5 (25–46.9), while the mean postoperative HHS was 71.7 (65.9–81.1). One study evaluated the modified D'Aubigne and Postel score, reporting an improvement in the pain subscale from a mean of 2.4 to a mean of 5.3 and in the walking subscale from a mean of 2.6 to a mean of 4.9 [8] (Table 3).

### Analysis of comparative studies

Five studies included in this systematic review were comparative [9, 15–18]. Three studies compared a group of patients with ETO PJI with a group of patients with non-ETO PJI. The most outstanding result was that in two of them, the rate of re-debridement for persistent signs of infection was lower for the ETO PJI group [15, 16], and in one study [15], the reinfection rate was lower in the ETO PJI group. No significant differences were reported for the other variables evaluated. Two studies [9, 18] compared a group of ETO PJI patients with non-PJI ETO patients. The most important result was the absence of significant differences in postoperative complications, both septic and aseptic, and for postoperative HHS. Table 4 summarizes the results of the comparative studies analyzed.

**Table 4:** Summary of findings of comparative studies between ETO PJI versus non-ETO PJI, and ETO PJI versus non-PJI ETO patients

Findings*	ETO PJI versus non-ETO PJI patients			ETO PJI versus non-PJI ETO patients	
	Hardt et al. 2021 [16]	Lancaster et al. 2021 [17]	Shi et al. 2019 [15]	Whittaker et al. 2022 [18]	Lim et al. 2011 [9]
Reinfection rate	=	=	↑	=	N/A
ETO union rate	N/A	N/A	N/A	=	=
Re-debridement	↑	N/A	↑	N/A	N/A
Femoral stem subsidence	=	=	=	=	=
Dislocation	=	=	=	=	=
Periprosthetic fracture	=	=	=	=	=
Postop HHS	=	N/A	=	N/A	=
Follow-up	=	=	=	=	=

ETO: Extended Trochanteric Osteotomy; PJI: periprosthetic joint infection; Harris Hip Score: HHS; N/A: not available; ↑: Better outcomes in ETO-PJI patients; = : No differences between groups of patients; *: No worse outcome were reported in ETO-PJI patients

## Discussion

The most important finding of this systematic review was that ETO, in two-stage hip revision in PJI, demonstrated excellent clinical outcomes providing an optimal osteotomy flap healing rate (94.8%), low recurrent infection incidence (8.9%), and femoral component subsidence greater than 5 mm (4.1%). Despite these results, a high postoperative aseptic complications rate of 17.7%, mainly represented by recurrent dislocations, periprosthetic fractures, and stem subsidence higher than 5 mm, was reported [7–9, 13–16, 18]. Current literature established that ETO fixed with cerclages may be a safe procedure in patients with a stable and well-fixed femoral stem that underwent two-stage hip revision [18]. Furthermore, other devices, such as trochanteric plates, were discouraged due to potentially increasing infection recurrence risk [7, 16].

Another important result was the lower re-debridement rate in patients who underwent the ETO procedure compared to non-ETO [15, 16]. This finding could be related to better femoral canal and acetabulum visualization, ensuring complete and thorough debridement and accurate cement removal [7, 8].

Regarding PROMs, the studies included in this systematic review demonstrated that ETO was an effective procedure that did not affect patients' long-term clinical and functional outcomes. Three studies analyzed two groups of patients, ETO and non-ETO, with hip PJI. Compared with preoperative one, postoperative HHS improved in all the patients, but no statistically significant differences were reported between ETO and non-ETO groups [13, 15, 16]. Lim et al. compared patients undergoing ETO procedures for hip PJI or revision aseptic THA. Similarly, no statistically significant differences were described between the two groups analyzed [9].

### Osteotomy flap healing

The ETO healing rate in PJI revisions was 94.8% and occurred in an average of 12 weeks [9, 13–16, 18]. These results were comparable to Malahias et al.'s systematic review, where an ETO healing rate of 93.1% was described in aseptic hip revisions [6]. Two studies that compared one group of ETO-PJI procedures with another of ETO in the aseptic hip revision reported no significant difference in the overall ETO union rate [9, 18]. In addition, Whittaker et al., in their cohort of 49 patients, examined possible variations between wires or cables flap fixation, reporting no differences in union and reinfection rates and no cases of osteotomy flap non-union [18]. Lim et al. [19] observed a higher ETO healing rate, although not statistically significant, in the PJI group than in the aseptic revision one, describing healing rates of 100% and 98%, respectively.

### Reinfection

The overall reinfection rate of patients undergoing ETO for hip PJI was 8.9% significantly higher than the 1.5–2% PJI rate after primary THA [28, 29] but comparable to the overall reinfection rate of 8.4% reported in the literature after two-stage hip revision for PJI [30]. Among the studies included in this systematic review, Morshed et al. reported the highest reinfection rate (three cases, 32.1%) [7]. Two patients were being treated with immunosuppressive therapy and underwent further two-stage revision, reporting no clinical infection signs at the last follow-up; the third patient was affected by a persistent infection due to Candida albicans and died postoperatively for his poor clinical condition [7]. Levine et al. reported a case in which recurrent infection occurred a few weeks after reimplantation [8]. This patient developed non-union ETO that was treated with an allograft composite transplant, wire removal, and chronic suppressive antibiotic therapy.

## Dislocation

The postoperative recurrent dislocation rate in patients undergoing ETO for PJI was 8.2%. Morshed et al. [7] reported the highest dislocation rate of 31% of cases, while an incidence higher than 10% was reported by Hardt et al. [16] and Whittaker et al. [18]. Although patients who underwent ETO in a two-stage hip revision had a higher dislocation frequency than primary THA patients, there were no significant differences in dislocation rate, about 10% according to the most recent literature and registry data, between ETO-PJI procedures and non-ETO THA septic or aseptic revision [31–34]. Several risk factors that could increase postoperative hip instability were described in the included studies. Morshed et al. [7] reported an important correlation between the recurrent dislocation rate and a severe bone defect of at least Paprosky type III and IV for the acetabular side and Paprosky type III for the femoral component. The abductor muscles' deficiency or weakness, often compromised after multiple surgeries, is another significant risk factor for the recurrent dislocation rate [8]. Conversely, as reported in several studies [27, 28], adequate soft tissue tensioning and sparing may be a protective factor against postoperative hip instability. Lim et al. [9] suggest using articulating spacers instead of cement beads to reduce postoperative dislocation rates.

### Limitations

This systematic review had some limitations that need to be discussed. First, the study quality was relatively low, as reflected by the mean MINORS score. A meta-analysis was not performed because there were no level I or II studies. The total number of patients included was quite small due to the rarity of the ETO procedure in PJI. Third, ETO techniques and fixations methods were different among the included studies, which may influence the outcomes analyzed. Further studies with a larger patient sample and homogeneity of techniques for performing ETO and fragment fixation could improve data validity.

## Conclusions

ETO in two-stage hip revision is a safe and successful procedure, with a reinfection rate of 8.9%, comparable with two-stage hip revision without ETO procedure, and a fragment healing rate of 94.8% in line with the ETO healing rate in non-septic hip revision. These results suggest that ETO could be safely performed in hip PJI with a stable femoral component to reduce intra-operative fractures, characterized by worse clinical results, during stem and cement removal.

## Data Availability

The dataset analyzed in this study is available from the corresponding author on reasonable request.
